# CarrotDB: a genomic and transcriptomic database for carrot

**DOI:** 10.1093/database/bau096

**Published:** 2014-09-27

**Authors:** Zhi-Sheng Xu, Hua-Wei Tan, Feng Wang, Xi-Lin Hou, Ai-Sheng Xiong

**Affiliations:** State Key Laboratory of Crop Genetics and Germplasm Enhancement, College of Horticulture, Nanjing Agricultural University, Nanjing 210095, China

## Abstract

Carrot (*Daucus carota* L.) is an economically important vegetable worldwide and is the largest source of carotenoids and provitamin A in the human diet. Given the importance of this vegetable to humans, research and breeding communities on carrot should obtain useful genomic and transcriptomic information. The first whole-genome sequences of ‘DC-27’ carrot were *de novo* assembled and analyzed. Transcriptomic sequences of 14 carrot genotypes were downloaded from the Sequence Read Archive (SRA) database of National Center for Biotechnology Information (NCBI) and mapped to the whole-genome sequence before assembly. Based on these data sets, the first Web-based genomic and transcriptomic database for *D. carota* (CarrotDB) was developed (database homepage: http://apiaceae.njau.edu.cn/car rotdb). CarrotDB offers the tools of Genome Map and Basic Local Alignment Search Tool. Using these tools, users can search certain target genes and simple sequence repeats along with designed primers of ‘DC-27’. Assembled transcriptomic sequences along with fragments per kilobase of transcript sequence per millions base pairs sequenced information (FPKM) information of 14 carrot genotypes are also provided. Users can download *de novo* assembled whole-genome sequences, putative gene sequences and putative protein sequences of ‘DC-27’. Users can also download transcriptome sequence assemblies of 14 carrot genotypes along with their FPKM information. A total of 2826 transcription factor (TF) genes classified into 57 families were identified in the entire genome sequences. These TF genes were embedded in CarrotDB as an interface. The ‘GERMPLASM’ part of CarrotDB also offers taproot photos of 45 carrot genotypes and a table containing accession numbers, names, countries of origin and colors of cortex, phloem and xylem parts of taproots corresponding to each carrot genotype. CarrotDB will be continuously updated with new information.

**Database URL:** http://apiaceae.njau.edu.cn/carrotdb/

## Introduction

Carrot (*Daucus carota* L.) species belong to the family Apiaceae, which comprises up to 3700 species across 450 genera worldwide (1, 2). Carrot is among the top 10 vegetable crop members in terms of production area and market value with >20 million tons of production a year worldwide according to data from the Food and Agriculture Organization of the United Nations (http://fao stat3.fao.org). Most cultivated carrots are functional diploids (2n = 2× = 18) with a genome size of ∼480 Mb (3). The modern cultivated carrot genus (*D. carota* spp. *sativus*) is genetically diverse and is classified into two groups, namely, carotene (*D. carota* ssp. *sativus* var. *sativus*) and anthocyanin groups (*D. carota* ssp. *sativus* var. *atrorubens* Alef.) (4). Carotene carrot cultivars are an important source of carotenoids and provitamin A and account for the majority of the species of carrot. Carotene carrot cultivars have been cultivated as root crops for ∼1100 years, whereas anthocyanin group carrots have a history dating back 3000 years (3, 5). Over the centuries, carrot transformed from being a small, tough and bitter root to a fleshy, sweet, pigmented, unbranched and edible vegetable.

In recent years, with increasing number of genomic and genetic studies on carrot, some carrot data sets have become publicly available. *De novo* assembly of the inbred carrot line ‘B493B’ mitochondrial genome has been reported (6). This assembly provided new information on intercompartmental interactions and cell evolution in this species. Transcriptomic sequences of three carrot lines have also been *de novo* assembled (3), and these sequences revealed new information, such as novel genes and markers. RNA-seq raw data sets of 11 carrot genotypes have been uploaded to the NCBI SRA database (http://www.ncbi.nlm.nih.gov/sra/). Many expressed sequence tags (ESTs) are also available on NCBI. These data are useful for genomic and transcriptomic analysis. However, no database for carrot is currently available despite that carrot is among the top 10 important vegetable crops worldwide. Given the importance of carrot to humans, a carrot genomic and transcriptomic database should be available for researchers to explore important agronomic characteristics of carrots, such as contents of β-carotene, anthocyanins or sugar; resistance to disease; and taproot tolerance to abiotic stress. Based on the completion of the whole-genome sequence of an inbred line ‘DC-27’ (*D. carota* ssp. *sativus* L.) in our laboratory using next-generation sequencing, a genomic and transcriptomic database for *D. carota* (CarrotDB) has been constructed for the use of the global scientific community. CarrotDB is now publicly available (http://apiaceae.njau.edu.cn/carrotdb).

In this Web site, the *de novo* assembled draft genome sequence of ‘DC-27’ is available. Users can also search the transcriptomic sequences of 14 carrot genotypes and their expected number of fragments per kilobase of transcript sequence per millions base pairs sequenced information (FPKM). The transcriptome sequences have been assembled by mapping reads to whole-genome sequences. Based on these resources, easy-to-use interfaces were also developed to help researchers find sequences of the scaffolds, putative genes or gene fragments, simple sequence repeat (SSR) markers, expressed genes in the transcriptome and FPKM information of expressed genes by keyword search or homology search. Researchers can check gene annotation and submit information to the GENOME MAP group. This database will be useful for scientists worldwide who are working on carrot genetics.

### Data resources

The data sets of information in the current CarrotDB version were based on the analysis of the genome sequence of ‘DC-27’ (an F_8_ inbred line of a commercial variety ‘Kuroda’ from our laboratory) and transcriptome sequences of 14 carrot genotypes. The carrot materials were obtained from Lab of Apiaceae Plant Genetics and Germplasm Enhancement, State Key Laboratory of Crop Genetics and Germplasm Enhancement, Nanjing Agricultural University. We provided the whole draft genome sequences, nucleotide sequences of putative genes and amino acid sequences of putative proteins. We also provided SSRs and designed primers of ‘DC-27’ carrot. We assembled transcriptomic sequences along with FPKM information of 14 carrot genotypes in CarrotDB.

#### Genome sequencing and *de novo* assembly

**Figure 1.**
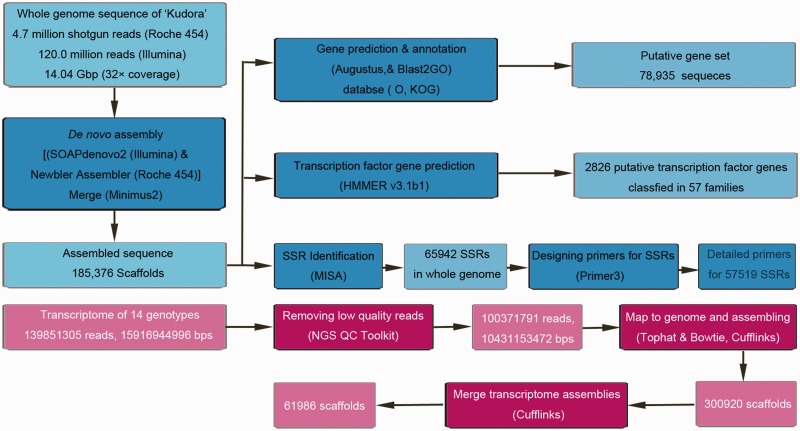
Workflow for processing and analyzing of genomic sequences of ‘DC-27’ and transcriptomic sequences specific to 14 carrot genotypes.

DNA was extracted from ‘DC-27’ carrot and sequenced using Roche 454GS FLX^+^ Titanium sequencer and Illumina HiSeq 2000 (CA, USA). We obtained ∼14.04 Gb of raw data. The 4.7 million shotgun reads and 120.0 million raw reads reached up to 32× coverage of the genome sequence, and these reads were generated in Roche 454 sequencing and HiSeq 2000 sequencing, respectively. SOAPdenovo software (http://soap.genomics.org.cn/soap denovo.html) (7) and Newbler Assembler software (http://swes.cals.arizona.edu/maier_lab/kartchner/documentation/index.php/home/docs/newbler) were used for assembly. After assembly, merging with Minimus2 software (http://sourceforge.net/apps/mediawiki/amos/index.php?title =Mi ni mus2) was performed. An assembly of 371.6 Mb was produced. Details of assembly procedure are shown in Figure 1.

#### Gene prediction, annotation and similarity analysis

Using Augustus software, 78 935 putative genes or gene fragments were predicted (8). The total, average and maximum lengths were 65.8 Mb, 833 bp and 8217 bp, respectively. These putative genes were annotated using Blast2GO with Gene Ontology and Eukaryotic Clusters of Orthologous Groups databases (9–11).

#### Identification of transcription factor genes

Transcription factors (TFs) play a vital role in regulating gene expression and plant growth development. We used HMMER (v3.1b1; cutoff *E*-value:1e-5) to predict TFs (12). A total of 2826 TF genes were identified in the whole-genome sequences. These putative TFs genes were classified into 57 families. *MYB* gene family was the largest among all these predicted TF families, followed by *ABI3-VP1* and *AP2-EREBP* gene families. *MYB* gene family contained 386 gene members.

#### Development of genome-wide SSR markers

SSR markers are among the most important molecular markers in plants and have been widely used in carrots (3, 13–17). Based on the draft genome sequence of ‘DC-27’, SSRs were identified using MIcroSAtellite (MISA) identification tool (http://pgrc.ipk-gatersle ben.de/misa/). A certain minimum number of units were 10, 6, 5, 5, 5 or 5 for mono-, di-, tri-, tetra-, penta- or hexanucleotide repeats, respectively. A total of 65 942 SSRs were found in the whole-genome sequence of ‘DC-27’, including 48 398, 15 172, 2316, 4, 8 and 44 for mono-, di-, tri-, tetra-, penta- and hexanucleotide repeats, respectively (Table 1). To develop SSR markers, 57 519 pairs of primers specific to SSRs were designed using Primer3 software (18).

**Table 1.** Identified SSRs in whole genome sequence of ‘DC-27’

Repeat type of SSR	Minimum units	Quantity
Mononucleotide repeats	10	48 398
Dinucleotide repeats	6	15 172
Trinucleotide repeats	5	2316
Tetranucleotide repeats	5	4
Pentanucleotide repeats	5	8
Hexanucleotide repeats	5	44
Total		65 942

### Assembly of transcriptomic sequences of 14 carrot genotypes

The RNA-seq raw data of 14 carrot genotypes were downloaded from the NCBI SRA database. The variety names of carrots and accession numbers of data sets specific to the carrot genotypes are listed in Table 2. The raw read sets of ‘B493xQAL’, ‘B6274’ and ‘B7262’ have been *de novo* assembled previously (3), whereas the raw read sets of other 11 carrot genotypes have not been assembled. Based on the whole-genome information of ‘DC-27’, we assembled or reassembled these read sets to obtain more accurate transcriptome assemblies. NGS QC Toolkit (v2.3.2) was used to remove reads with 10% or more low-quality bases (Phred score <20) (19). High-quality reads of 14 carrot genotypes were then mapped to the genome sequences of ‘DC-27’ with TopHat (v2.0.10) and Bowtie2 (2.1.0), separately, followed by assembly with cufflinks (20, 21). The nine resulting assemblies corresponding to ‘B493xQAL’, ‘B6274’ and ‘B7262’ were merged to three assemblies according to genotype. A total of 14 assemblies within each genotype containing thousands of contigs were generated. The quantities of scaffolds in each assembly are listed in Table 2. Resulting scaffolds in each genotype transcriptome assembly were separately used for Basic Local Alignment Search Tool (BLAST) searches and annotation against nucleotide (NT), non-redundant (NR), Swiss-Prot and Cluster of Orthologous Groups of proteins (COG) databases using BLAST software (22). A total of 283 469 contigs, which were mapped to the genome sequence, were produced for 14 carrot genotypes with the maximum contig length of 26 633 bp for ‘B493xQAL’. The FPKM information of each contig was also generated in the assemblies. To generate non-redundant contig assembly, the resulting 14 assemblies within each genotype were merged to one assembly by using Cufflinks (v2.1.1) (23). A total of 61 986 contigs were generated with the maximum contig length of 26 726 bp. Details of assembly procedure are shown in Figure 1.

**Table 2.** Process and analysis of transcriptomes of 14 carrot genotypes

Genotype of carrot	Accession	Reads	Bases (bp)	HQ reads^a^	HQ bases (bp)	Number of contigs	Bases (bp)
B493xQAL	SRR187755	7103325	7.17E+08	1398708	141269508	61986	97732153
	SRR187756	13088922	1.6E+09	9185857	1120674554		
	SRR187757	10685104	6.52E+08	9966187	607937407		
	SRR187758	7533218	7.61E+08	1538700	155408700		
B6274	SRR187759	11310004	1.38E+09	8197365	1000078530	48499	90887030
	SRR187760	9883580	6.03E+08	9228881	562961741		
	SRR187761	7899211	7.98E+08	776162	78392362		
B7262	SRR187762	13643281	1.66E+09	9384251	144878622	46229	85503687
	SRR187763	11918024	7.27E+08	11065168	674975248		
Wild carrots from Lachish, Israel	ERR185929	676811	1.02E+08	594771	89215650	2264	1561741
Chantenay	ERR185930	5484558	8.23E+08	4538559	680783850	30918	34748896
Flakkee	ERR185931	5986979	8.98E+08	4937523	740628450	13930	12793328
Wild carrots from Meijendel, Netherlands	ERR185932	68669	10300350	61360	9204000	159	60288
Berlikumer	ERR185933	1235784	1.85E+08	1046645	156996750	3629	2787541
Amsterdamse Bak	ERR185934	1887061	2.83E+08	1570853	235627950	3673	2438495
Wild carrots from Esposende, Portugal	ERR185935	5842774	8.76E+08	4956301	743445150	16251	17443806
Parijse	ERR185936	7843337	1.18E+09	6609671	991450650	21830	25778255
Wild carrots from Schermer Polder, Netherlands	ERR185937	8353398	1.25E+09	7363816	1104572400	21712	20885790
Wild carrots from Trencin, Slovakia	ERR185938	4176206	6.26E+08	3642797	546419550	15305	15209873
Nantes	ERR185939	5231059	7.85E+08	4308216	646232400	14535	15628203

^a^High-quality (HQ) reads and bases were obtained after removing reads with 10% or more low-quality bases (PHRED score <20).

#### Identification of structural genes involved in anthocyanin biosynthesis from whole-genome and merge transcriptomic sequence assemblies

Anthocyanins are abundant in taproots of purple carrot cultivars, whereas cyanidin-based anthocyanins represent almost all of the anthocyanins in carrots (24). To detect the quality of transcriptomes assembled in this study, we searched the structural genes involved in cyanidin-based anthocyanin biosynthesis pathway. All the candidate structural genes, including 17 genes in 10 gene families, were involved in the cyanidin-based anthocyanin pathway. These genes were found in the whole draft genome sequence assembly and the merge transcriptome assembly using BLASTN (Table 3). Chalcone–flavonone isomerase *(CHI)*, flavonoid 3′-monooxygenase *(F3’H)* and UDP-galactose: flavonoid 3-O-galactosyltransferase (*UF3GaT*) gene sequences were identified in carrot for the first time. These sequences have not been reported in previous *de novo* assembled transcripts (3). These results emphasize the accuracy and depth of our genome and transcriptome database.

**Table 3.** Results of BLASTN of structural genes involved in anthocyanins biosynthesis in carrot genome and transcriptome assemblies

Gene family	Reference sequence source	GenBank ID	Scaffold IDs in genome sequence with hits to reference	Putative gene IDs in genome sequence with hits to reference	Contigs in merged transcriptome sequencewith hits to reference
*PAL*	*D. carota*	D85850.1	229	433	00412, 00410
	*D. carota*	AB089813.1	16648, 16649, 169243	17456, 17457, 76158	17301, 17302, 59170
	*D. carota*	AB435640.1	16648, 16649, 169243	17456, 17457, 76158	17301, 17302, 59170
*CA4H*	*Ammi majus*	AY219918.1	25749	24733	24075, 33981
*4CL*	*Petroselinum crispum*	X13324.1	1380	1988	02081
	*P. crispum*	X13325.1	1380	1988	02081
*CHS*	*D. carota*	AJ006779.1	27333, 21408	25908, 21339	20975, 25160
	*D. carota*	D16255.1	139899, 4932	61298, 6227	06288, 48099
	*D. carota*	D16256.1	139899, 4932	61298, 6227	06288, 48099
*CHI*	*Camellia nitidissima*	HQ269805.1	8876	10389	10363
*F3H*	*D. carota*	AF184270.1	17226	17950	17727
*F3’H*	*Petunia x hybrida*	AF155332.1	36531	31974	30143
*DFR*	*D. carota*	AF184271.1	1955, 1956, 35796	31520, 2745, 3152	02876, 29801, 29802
	*D. carota*	AF184272.1	44046	36059	33353
*LDOX*	*D. carota*	AF184273.1	20348, 182749, 20349, 20350	20467, 78025, 20472	20139, 20140, 20142
	*D. carota*	AF184274.1	20348, 182749, 20349, 20350	20467, 78025, 20470, 20472	61071, 20140, 20142
*UF3GaT*	*Vigna mungo*	AB009370.1	146346, 21238	64830, 21202	20841, 50490

*PAL*, Phenylalanine ammonia-lyase; *CA4H*, Cinnammate 4-hydroxylase; *4CL*, 4-coumaroyl-coenzyme A ligase; *CHS*, Chalcone synthase; *CHI*, Chalcone–flavanone isomerase; *F3H*, Flavanone 3-hydroxylase; *F3’H’*, Flavonoid 3’-monooxygenase; *DFR*, Dihydroflavonol 4-reductase; *LDOX*, Leucoanthocyanidin dioxygenase; *UF3GaT*, UDP-galactose: flavonoid 3-O-galactosyltransferase.

### Database construction

#### Database system implementation

**Figure 2.**
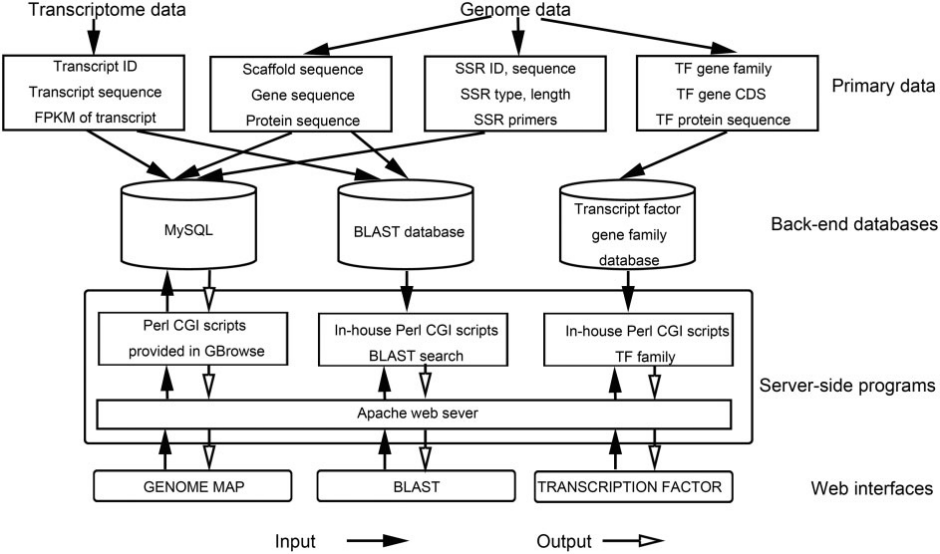
Overview of CarrotDB architecture.

A database system for integrating the produced carrot draft genome sequence, putative genes sequences, coding sequences, SSRs with specific primers, predicted TFs, transcript sequences and FPKM information was developed. An Apache HTTP server in a Linux (CentOS6.2) operating system was used to develop CarrotDB. We used PHP5, Perl scripts, HTML and JavaScript to build the user-friendly interface and to write the Web pages. Genome visualization was developed using the Generic Genome Browser (GBrowse1.7) package (25). The CarrotDB system also contains an installed BLAST server. An overview of the database architecture is shown in Figure 2.

## Genome map

**Figure 3.**
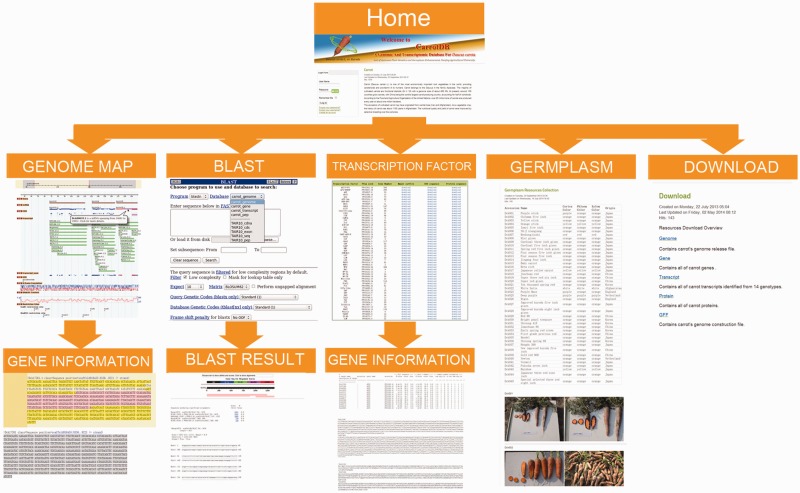
Organizational structure of CarrotDB Web pages.

In the genome map section, CarrotDB provides Genome Map tools to visualize the whole draft genome sequence of ‘DC-27’ using GBrowse (Figure 3). All 185 376 *de novo* assembled scaffolds were used to construct the genome browser. Five icons for each scaffold can be tracked to the detailed view: (i) gene, (ii) mRNA, (iii) coding sequences (CDS), (iv) transcript and (v) SSR. A total of 78 935 putative genes predicted from 185 376 assembled scaffolds of’DC-27’ are displayed here. Users can link to exons, introns and UTR sequences along with the locations of a putative gene on a selected assembled scaffold by clicking the gene icon. Clicking an mRNA icon leads users to the exons and UTR sequences along with the locations of a putative gene on a selected assembled scaffold. The CDS icon directs the user to the exon sequences along with the locations of a putative gene on a selected assembled scaffold. A total of 345 455 contigs from 14 assemblies corresponding to 14 carrot genotypes along with the merged assembles are also provided. Clicking on a transcript icon leads users to the transcript sequence along with the location on a selected assembled scaffold and FPKM information. Given the important uses of SSR markers in carrots, 65 942 SSRs are provided in this block. Clicking the SSR icon provides the link to the SSR sequence and primer sequences specific to SSRs. Users can freely obtain useful information from this section.

## BLAST

To help users perform sequence alignment, BLAST was embedded in the database using the wwwBlast program (v2.2.26) to provide a graphic user interface through the Web forms (Figure 3) (26). The whole draft genome sequence, putative gene sequences, protein sequences assemblies of ‘DC-27’, transcriptome sequences of 14 carrot genotypes and merged transcriptome sequences are used for BLAST. Users can perform similarity searches against each type of sequences using various BLAST search forms (BLASTn, BLASTp, BLASTx, tBLASTn and tBLASTx). The nucleotide acid or amino acid sequences in FASTA format can be submitted by entering the data in the frame or uploading a file. Users can set some suitable parameters or simply select the default parameters before performing the search. Clicking the search icon provides the link to the query results interface. Therefore, the query sequences along with their position on the whole-genome sequences of ‘DC-27’ were produced and ordered according to the expected value. This section also provides genome, gene and protein sequence databases of *Arabidopsis thaliana* for BLAST.

## Transcription factor

In plants, TFs control the transcription of downstream genes by binding to specific DNA sequences. For the users’ convenience, 2826 putative genes that have been classified into 57 TF families from the whole-genome sequence assembly of carrot ‘DC-27’ are listed in a table in this section. The quantities of putative TF genes in each family are shown in Figure 3. Clicking the download icon below the ‘Hmmer outfile’ will lead users to interfaces containing information from an HMMER outfile, such as target gene ID, expected value and HMMER score. Users can link to the interface of nucleotide sequences and deduced amino acid sequences of all the putative TF genes from each TF family by clicking the ‘download’ icons below **‘**CDS sequence’ or ‘Protein sequence’.

## Germplasm

Many genotypes of *D. carota* cultivars have been collected and stored in our laboratory. A total of 45 genotypes are displayed in this section (as shown in Figure 3). Every genotype of carrot has an accession number. The accession number, names, countries of origin and colors of cortex, phloem and xylem parts of taproots corresponding to each carrot genotype are listed in the table embedded in this section. The taproots of these carrots are in various colors, including orange, red, yellow, purple and white. These carrots also have different taproot shapes. We obtained some photos of the taproots of these carrots and provided the images in this interface.

## Download

Besides the BLAST section, the genome and transcriptome information of *D. carota* are also available (Figure 3) in this section. HTTP links are provided for users who want to freely download the released assembled genome sequences, nucleotide sequences of putative genes, gene annotation, amino acid sequences of putative proteins of ‘DC-27’ and transcriptome sequence assemblies of 14 carrot genotypes along with the merged transcriptome sequence assembly for research.

## Discussion

Carrot is among the top 10 economically important vegetable crops worldwide and is the most essential source of provitamin A carotenoids in the human diet. Given the importance of carrots to humans, a database containing the genome and transcriptome information of *D. carota*, CarrotDB, was developed. CarrotDB provides the whole draft genome sequences, nucleotide sequences of putative genes and amino acid sequences of putative proteins, and SSRs with the designed primers of ‘DC-27’ carrot, as well as assembled transcriptomic sequences with FPKM information of 14 carrot genotypes. We developed an easy-to-use Genome Map and BLAST tool interfaces for these data sets. These interfaces enable users to search and acquire target gene sequences efficiently. All the putative TF families were identified based on the whole draft genome sequence and were embedded in CarrotDB. We also provided photos of the taproots of 45 carrot genotypes. To our knowledge, CarrotDB is the first public genomic and transcriptomic database for carrot and for all members of the Apiaceae family.

The CDS of putative genes from the genome data set of ‘DC-27’ were computationally predicted and not experimentally proven. Whether these putative gene CDS are accurate or can produce alternative splices cannot be determined. Transcriptomes of three carrot genotypes were only *de novo* assembled in a previous work (3). Transcriptome sequence data sets are useful to improve accuracy of these putative gene sequences, whereas the whole genome sequence assembly is helpful for assembling transcriptome sequences. Transcriptomes of 14 carrot genotypes were assembled by mapping to the whole genome sequences, producing more accurate and deeper transcripts of carrots. The expression patterns of putative genes can be somehow represented by the FPKM information of transcripts. Researchers may also find gene alternative splicing in the transcripts of the 14 carrot genotypes.

SSR markers have been applied in many genomic research aspects, such as genetic polymorphism detection, genetic relationship determination, genetic map construction and gene localization. In carrots, SSRs have been developed based on EST sequences and are widely applied in genetic relationships and diversity assessment (13–16, 27), linkage mapping (15, 17) and gene location (28, 29). Based on the whole genome sequence, various genotypes of SSRs were identified in intergenic and intragenic regions. These SSRs along with the designed primers will be valuable for further research on carrots.

## Future directions

The current database version will be updated with new information. With increasing research on carrots, sequencing data will also emerge significantly. We will continuously collect more transcriptome data for further analysis and store such data in CarrotDB. Single-nucleotide polymorphisms in various genotypes of carrots will also be identified and released on the CarrotDB after we collect more transcriptome sequence data sets. Users are encouraged to send us feedback for further improvement of this database. We believe that CarrotDB will be a useful database for researchers and breeders who are working on carrots.

## Funding

The work was supported by the New Century Excellent Talents in University (NCET-11-0670); Jiangsu Natural Science Foundation (BK20130027); National Natural Science Foundation of China (31272175); China Postdoctoral Science Foundation (2014M551609); Priority Academic Program Development of Jiangsu Higher Education Institutions and Jiangsu Shuangchuang Project.

*Conflict of interest*. None declared.
